# Supplementary material for: Expanding PROTACtable genome universe of E3 ligases
Source: Nat Commun. 2023 Oct 16;14:6509. doi: 10.1038/s41467-023-42233-2 (PMC10579327; doi:10.1038/s41467-023-42233-2)
Supplement: Supplementary file 1 — Supplementary Information [file 41467_2023_42233_MOESM1_ESM.pdf]

Supplementary Information

Expanding PROTACtable genome universe of E3 ligases

Yuan Liu[1,2,3], Jingwen Yang[1,2,3], Tianlu Wang[4], Mei Luo[1,2], Yamei Chen[1,2,3], Chengxuan Chen[1,2,3], Ze'ev Ronai[5], Yubin Zhou[4,6], Eytan Ruppin[7,#], Leng Han[1,2,3,6,#]

Inventory of Supplementary Information

Supplementary Fig. 1 E3 ligases collected from three sources and score assigned. Outer ring representing
confidence score of E3 ligases. Inner ring representing E3 ligases collected from different E3 ligases sources. One slice representing 10 E3 ligases.

Supplementary Fig. 2 Dissection of the ligandability of E3 ligases. (a) Distribution of the number of categories of ligands identified for each E3 ligase. (b) Dissection of ligands of E3 ligases from each source (one bar representing 10 E3 ligases).

Supplementary Fig. 3 In silico prediction of drug-E3 ligase interactions. (a) AUC-ROC curve of HyperAttentionDTI in the cross-validation that both drugs and proteins in the testing set appear in the training set (b) AUC-ROC curve that drugs in the testing set don't appear in the training set. (c) AUC-ROC curve that proteins in the testing set don't appear in the training set. (d) AUC-ROC curve that both drugs and proteins in the testing set don't appear in the training set.

Supplementary Fig. 4 Expression landscape of co-opted E3 ligases in tumors. (a) Expression of E3 ligases in TCGA. Top barplot showing the number of highly expressed cancer types of the E3 ligases in TCGA. (b) Expression of E3 ligases in HPA pathology. Bottom barplot showing the number of highly expressed cancer types of the E3 ligases in HPA pathology.

Supplementary Fig. 5 Expression of E3 ligases in tumors at single-cell level. (a-d) Expression of E3 ligases in NSCLC, UVM, PAAD, Glioma cancer type at single cell level, respectively. Pie plot showing the distribution of cells expressing E3 ligases. Dot plot showing the percentage and level of cells expressed E3 ligases within the cell type.

Supplementary Fig. 6 Expression of co-opted E3 ligases in normal tissues. (a) Expression of E3 ligases in GTEx. Top barplot showing the number of high expression of tissues of the E3 ligases in GTEx. (b) Expression of E3 ligases in Tabula. Bottom barplot showing the number of high expression of tissues of the E3 ligases in Tabula.

Supplementary Fig. 7 Expression of potentially novel E3 ligases by cell type in tissues at single-cell level. (a-b) Dissection of expression of TRAF3 in colon and skin in Tabula. (c) Dissection of expression of CBL in liver in Tabula.

Supplementary Fig. 8 Expression of VHL by cell type in liver and thymus at single-cell level.

Supplementary Fig. 9 Logical relationship of E3 ligases highlighted in different expression atlas. Venn plot of E3 ligases that were highly expressed in at least one cancer type in TCGA and HPA pathology and lowly expressed in the majority number of normal tissues in GTEx and Tabula.

Supplementary Fig. 10 Dissection of expression of co-opted E3 ligases featured in four datasets by cancer type.

Supplementary Fig. 11 The distribution of the number of E3 ligases interacting with PROTAC tractable targets by E3 ligase type.

Supplementary Fig. 12 Functional essentiality of co-opted E3 ligases.

Supplementary Fig. 13 Number of PPI interfaces associated with co-opted E3 ligases.

Supplementary Fig. 14 Panels of E3Atlas web portal. (a) E3 ligase profile module: directly going into a specific gene's profile. (b) comprehensive profile page of E3 ligase: showing the profile of a specific E3 ligase. (c) General E3 ligase search module: offering users the to create a set of criteria and define their own threshold. (d) Search by target module: offering users to search for E3 ligases against a specific target. (e) Search results as a table.

Supplementary Fig. 15 Use case: Integration of ligandability, PPI, expression pattern, confidence score, and cellular location of E3 ligases. From outer to inner ring, PPI, ligandability, E3 ligases, the number of high expression of cancer types in TCGA, the number of high expression of normal tissues in GTEx, confidence score, and cellular localization. E3 ligases in red were proceeded to PROTAC clinical trials. E3 ligases in blue were experimentally explored in PROTAC experiment. E3 ligases in bold were mentioned in context.

Supplementary Fig. 16 Use Case: Searching for E3 ligases against a critical oncogene, KRAS. From top to bottom: cellular location, ligandability, real structure availability, number of PPIs, expression level in PAAD, COAD, LUAD, LUSC, CHOL, UCEC, TGCT, and CESC, percentage of highly expressed cancer type in TCGA, and percentage of highly expressed normal tissues in GTEx. E3 ligases in bold were mentioned in context.

Supplementary Fig. 17 Use Case: Searching for E3 ligases against a critical oncogene, EGFR. From top to bottom: cellular location, ligandability, essentiality, number of PPIs, expression level in COAD, LUAD, and LUSC, percentage of highly expressed cancer type in TCGA, and percentage of highly expressed normal tissues in GTEx. E3 ligases in bold were mentioned in context.

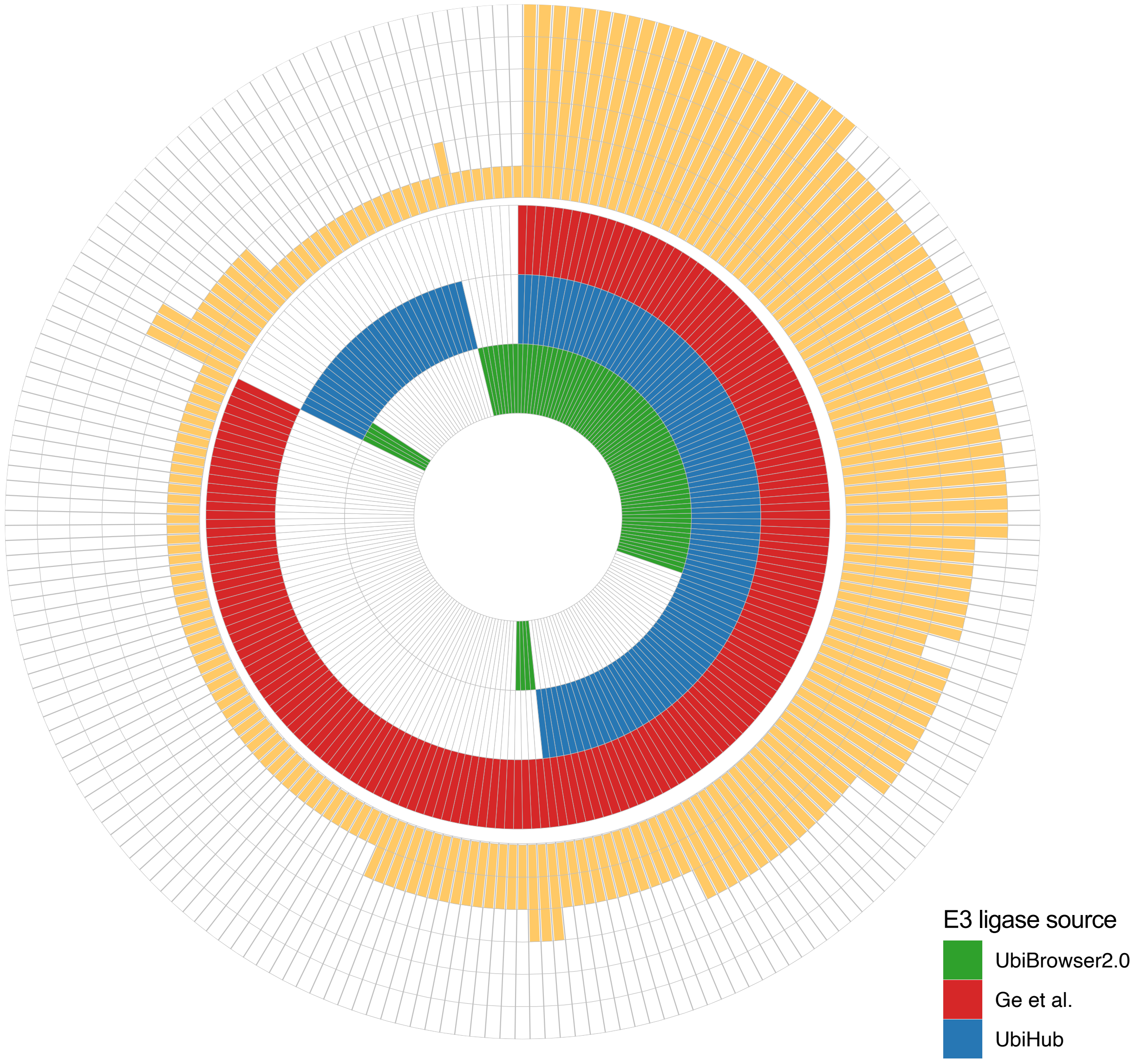

Supplementary Fig. 1 E3 ligases collected from three sources and score assigned. Outer ring representing confidence score of E3 ligases. Inner ring representing E3 ligases collected from different E3 ligases sourc- es. One slice representing 10 E3 ligases.

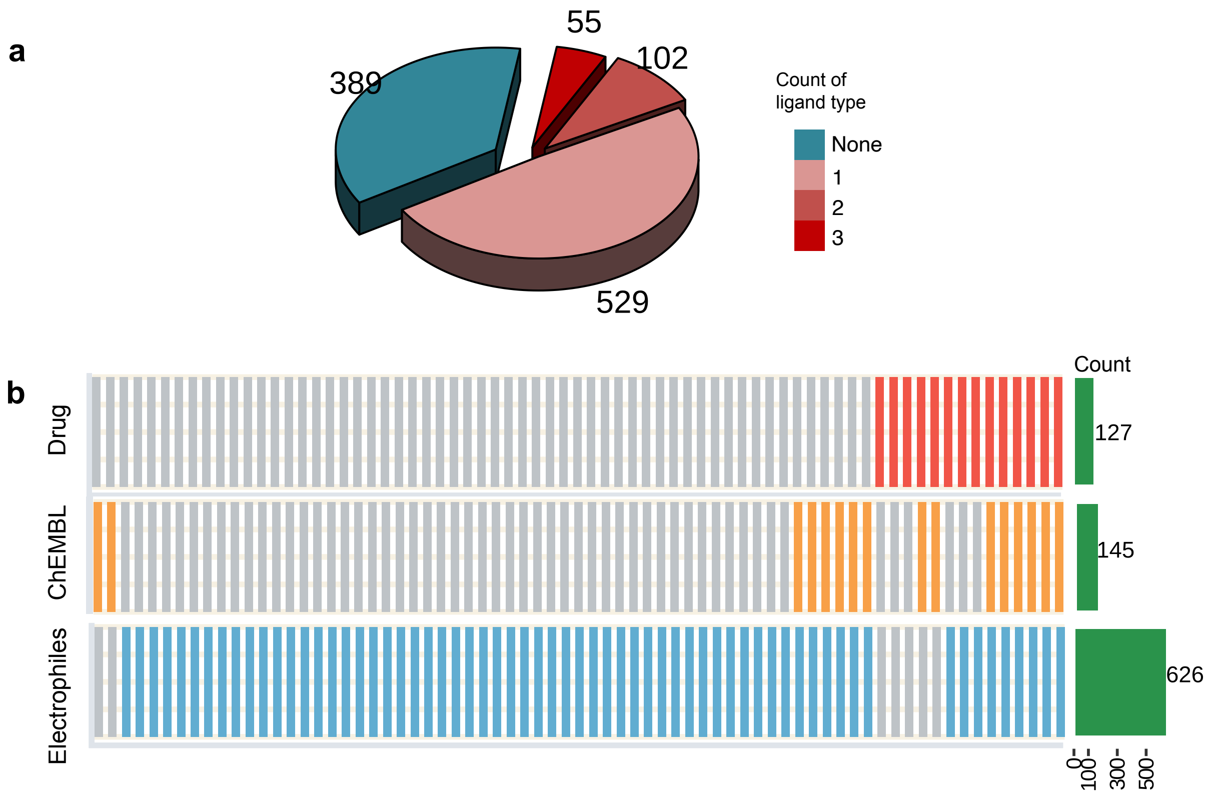

Supplementary Fig. 2 Dissection of the ligandability of E3 ligases. (a) Distribution of the number of sources of ligands identified for each E3 ligase. (b) Dissection of ligands of E3 ligases from each source (one bar representing 10 E3 ligases).

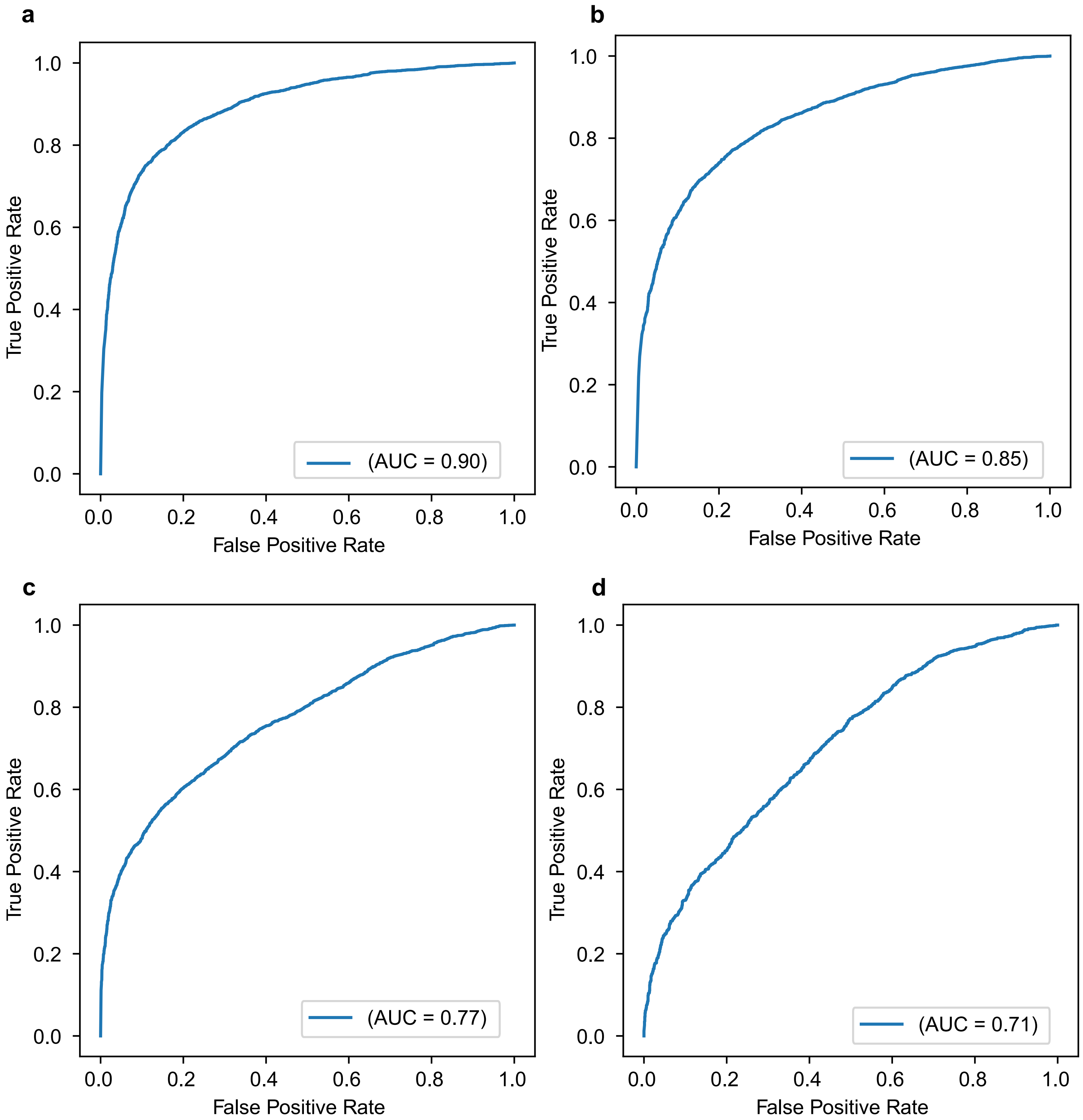

Supplementary Fig. 3 The evaluation of in silico prediction model of drug-E3 ligase interactions. (a) AUC-ROC curve of HyperAttentionDTI in the cross-validation that both drugs and proteins in the testing set appear in the training set (b) AUC-ROC curve that drugs in the testing set don't appear in the training set. (c) AUC-ROC curve that proteins in the testing set don't appear in the training set. (d) AUC-ROC curve that both drugs and proteins in the testing set don't appear in the training set.

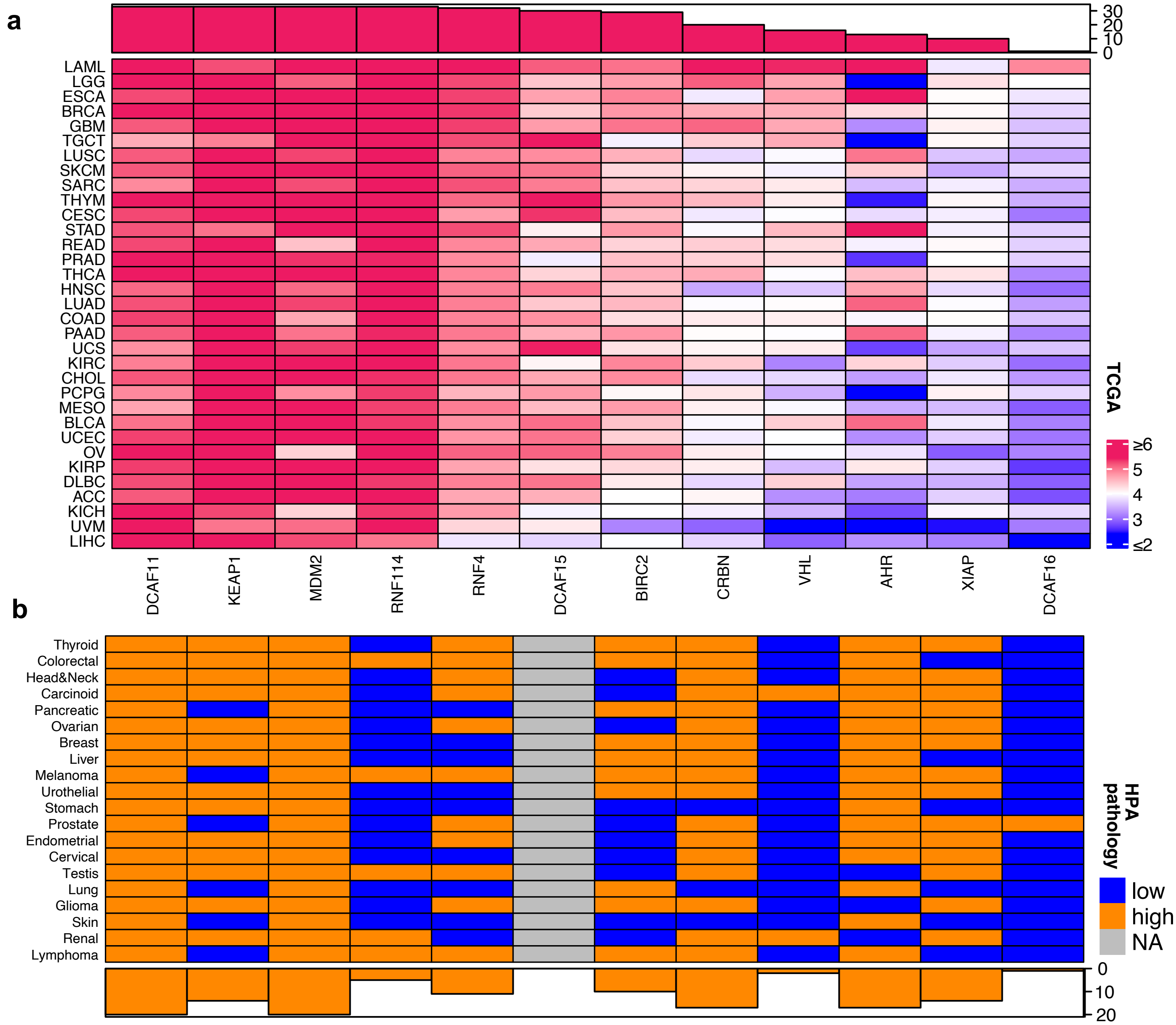

Supplementary Fig. 4 Expression landscape of co-opted E3 ligases in tumors. (a) Expression of E3 ligases in TCGA. Top barplot showing the number of highly expressed cancer types of the E3 ligases in TCGA. (b) Expression of E3 ligases in HPA pathology. Bottom barplot showing the number of highly expressed cancer types of the E3 ligases in HPA pathology.

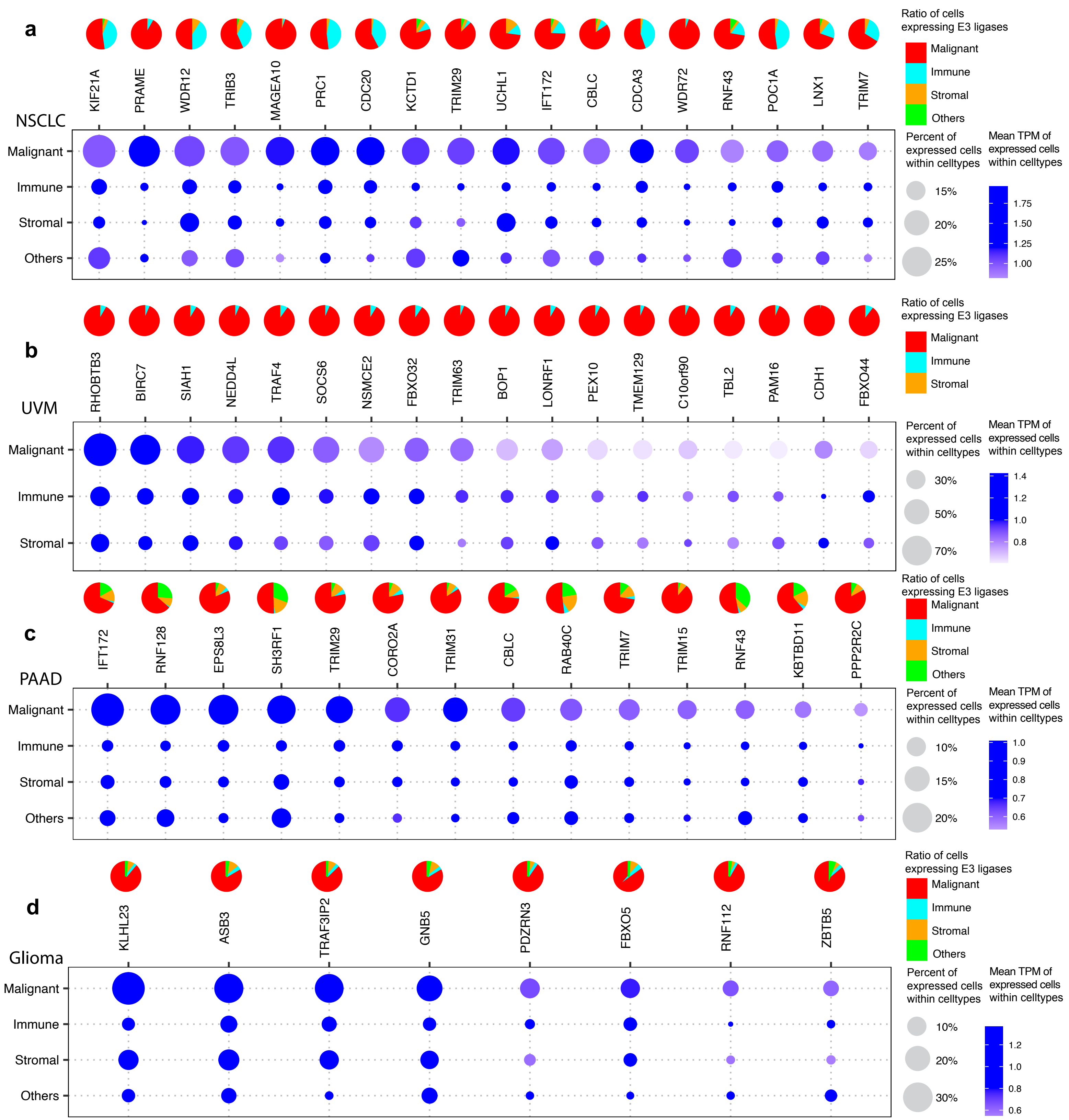

Supplementary Fig. 5 Expression landscape of co-opted E3 ligases in tumors. (a-d) Expression of E3 ligases in NSCLC, UVM, PAAD, Glioma cancer type at single cell level, respectively. Pie plot showing the distribution of cells expressing E3 ligases. Dot plot showing the percentage and level of cells expressed E3 ligases within the cell type.

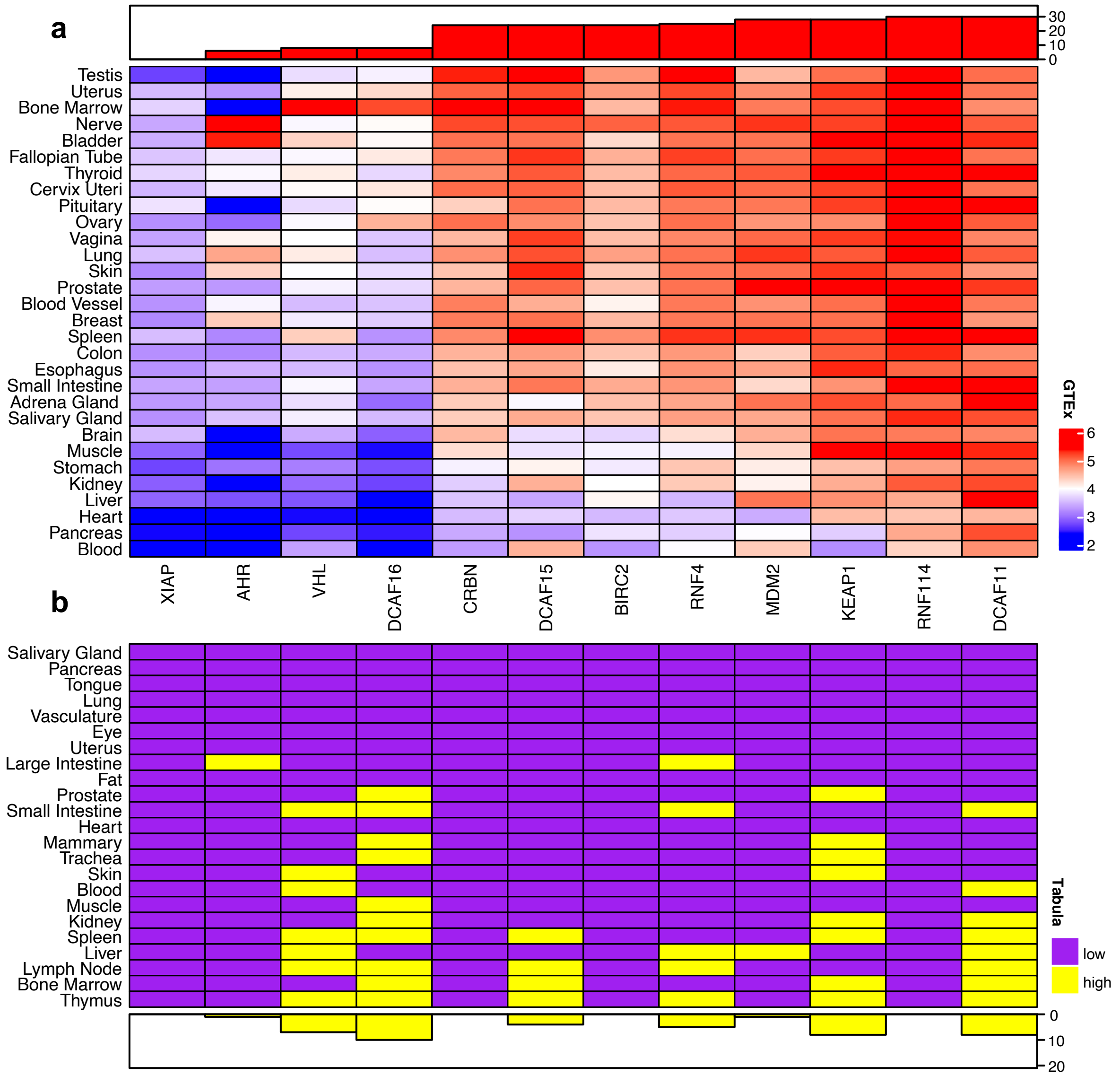

Supplementary Fig. 6 Expression of co-opted E3 ligases in normal tissues. (a) Expression of E3 ligases in GTEx. Top barplot showing the number of high expression of tissues of the E3 ligases in GTEx. (b) Expression of E3 ligases in Tabula. Bottom barplot showing the number of high expression of tissues of the E3 ligases in Tabula.

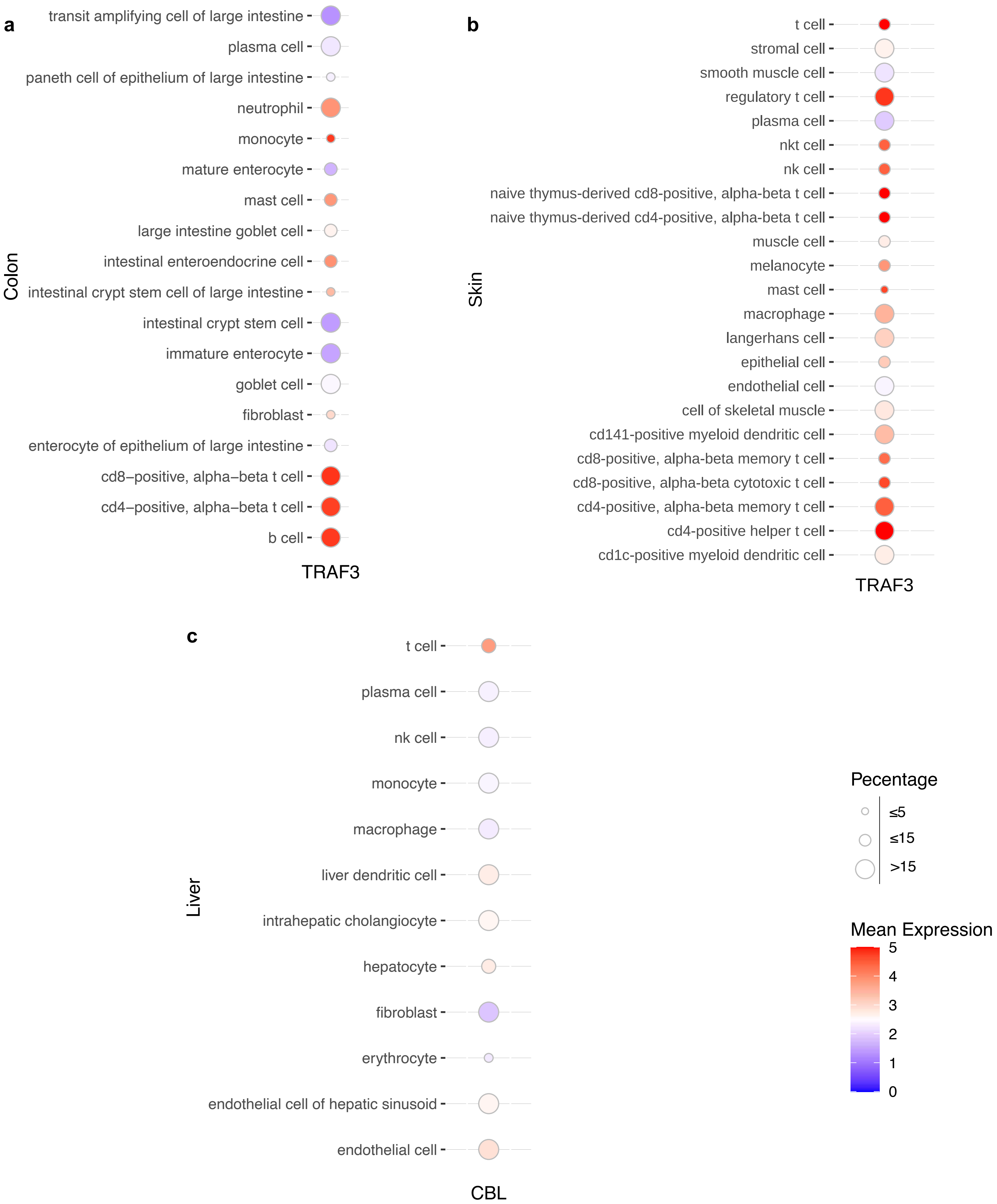

Supplementary Fig. 7 Expression of potentially novel E3 ligases by cell type in tissues at single-cell level. (a-b) Dissection of expression of TRAF3 in colon and skin in Tabula. (c) Dissection of expression of CBL in liver in Tabula.

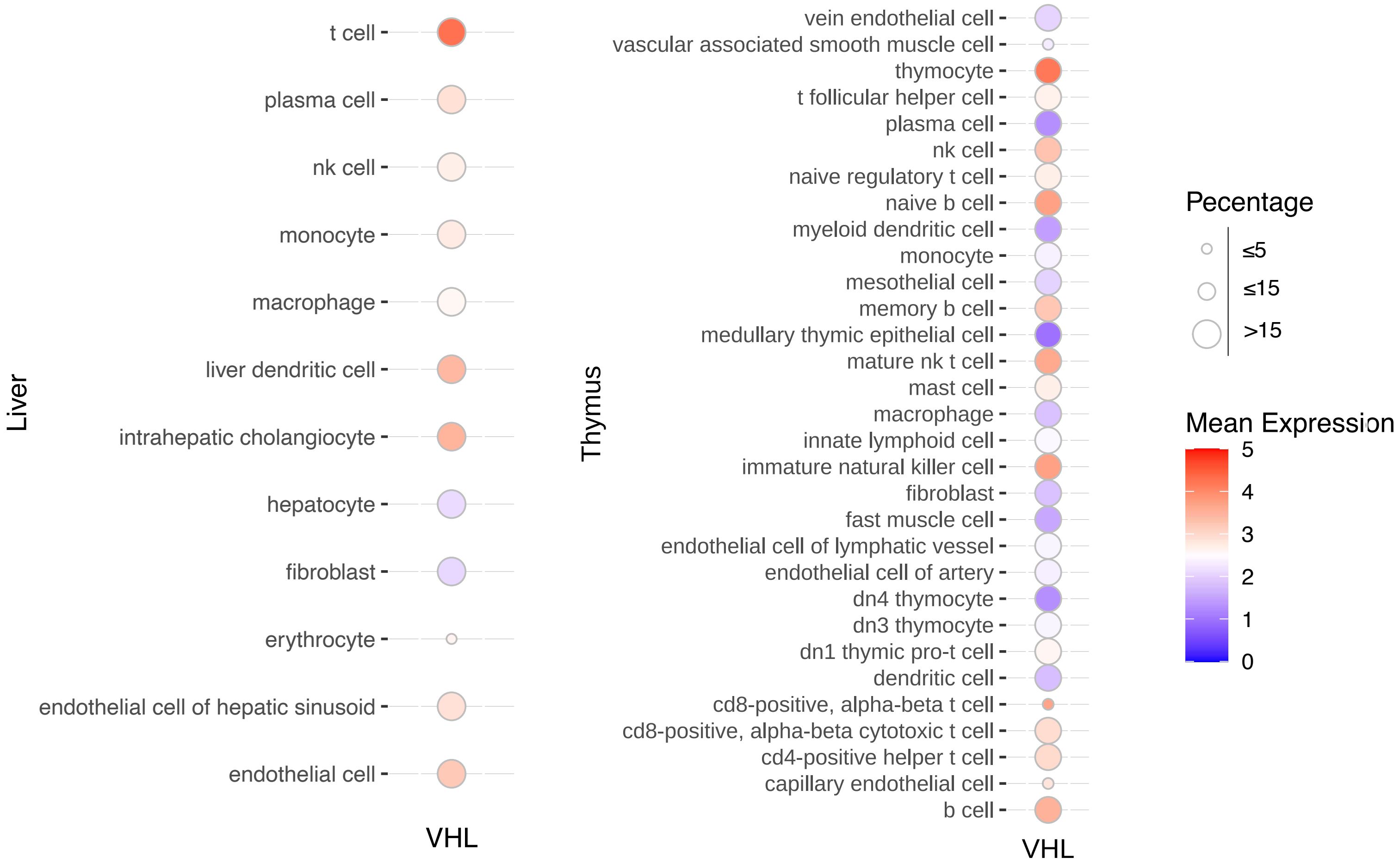

Supplementary Fig. 8 Expression of VHL by cell type in liver and thymus at single-cell level.

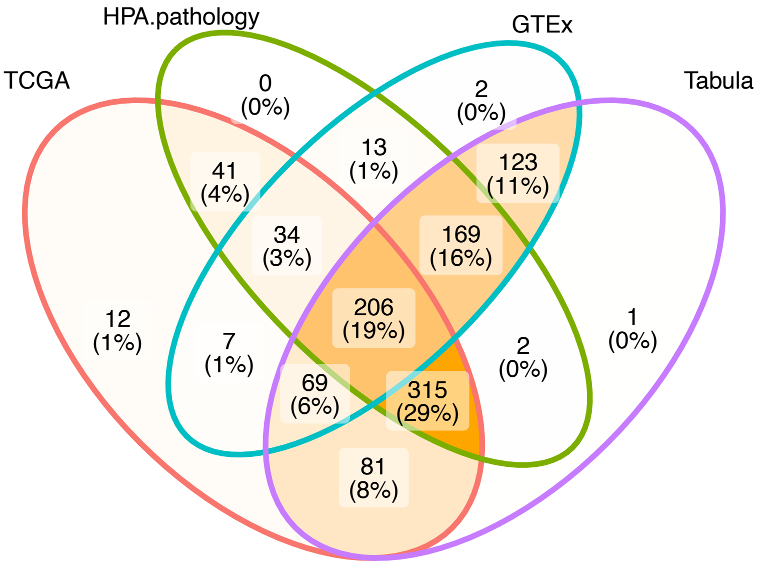

Supplementary Fig. 9 Logical relationship of E3 ligases highlighted in different expression atlas.

Venn plot of E3 ligases that were highly expressed in at least one cancer type in TCGA and HPA pathology and lowly expressed in the majority number of normal tissues in GTEx and Tabula.

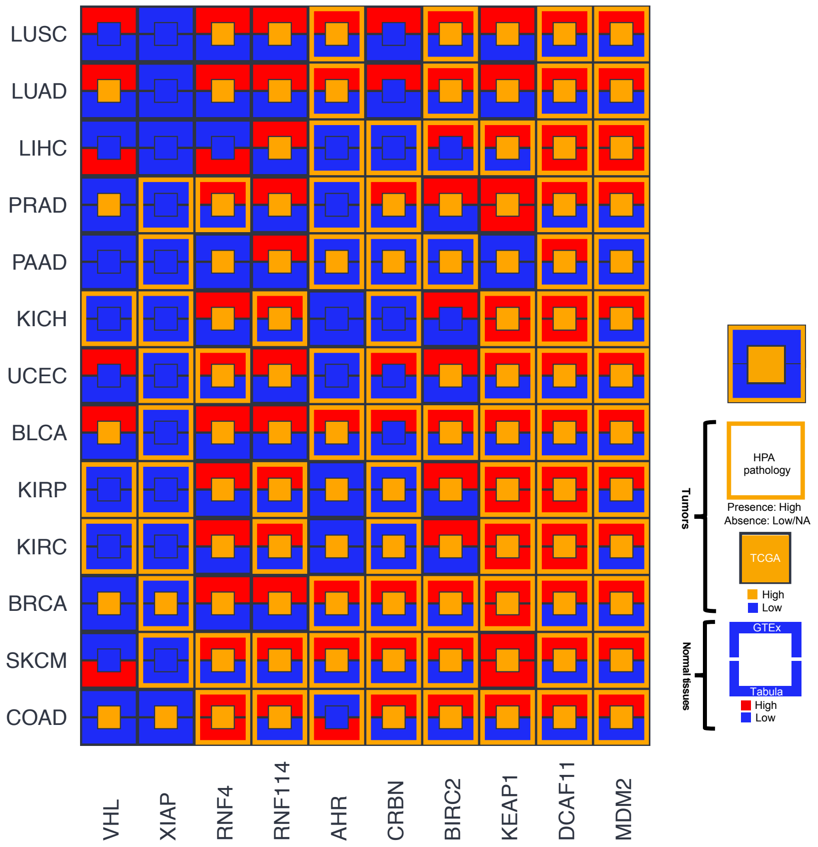

Supplementary Fig. 10 Dissection of expression of co-opted E3 ligases featured in four datasets by cancer type.

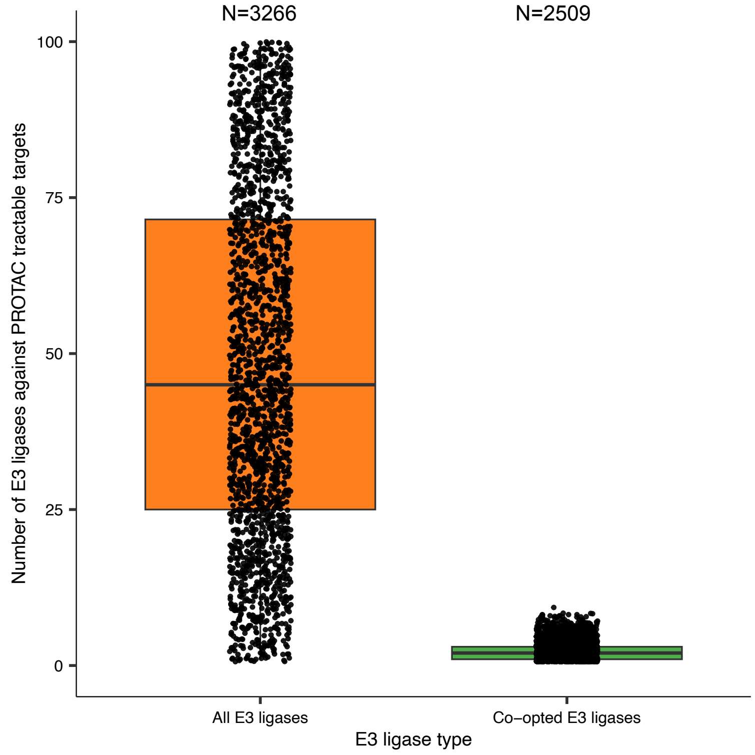

Supplementary Fig. 11 The distribution of the number of E3 ligases interacting with PROTAC tractable targets by E3 ligase type.

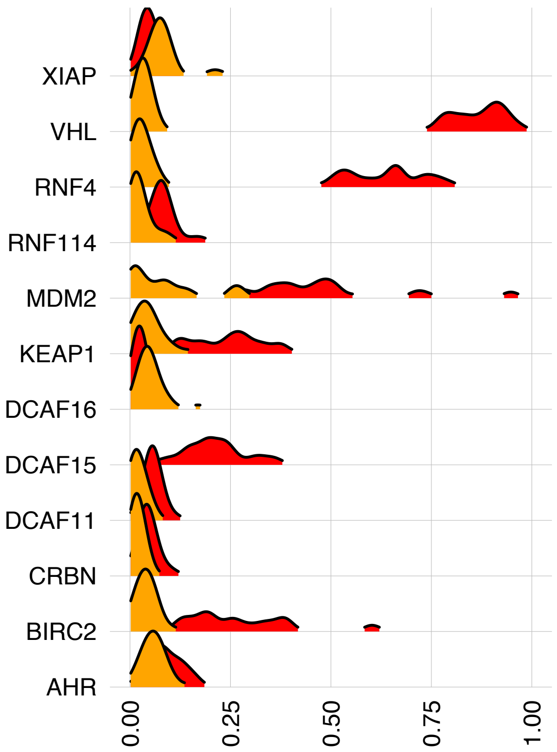

Supplementary Fig. 12 Functional essentiality of co-opted E3 ligases.

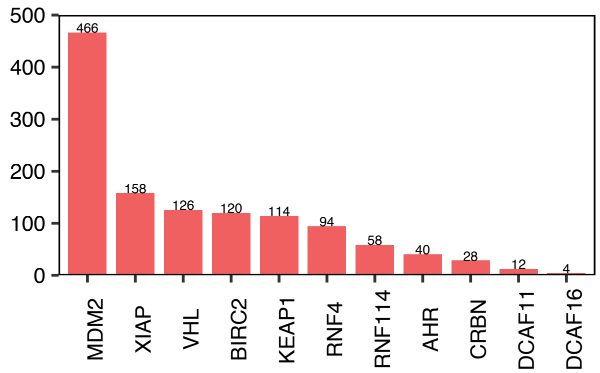

Supplementary Fig. 13 Number of PPI interfaces associated with co-opted E3 ligases

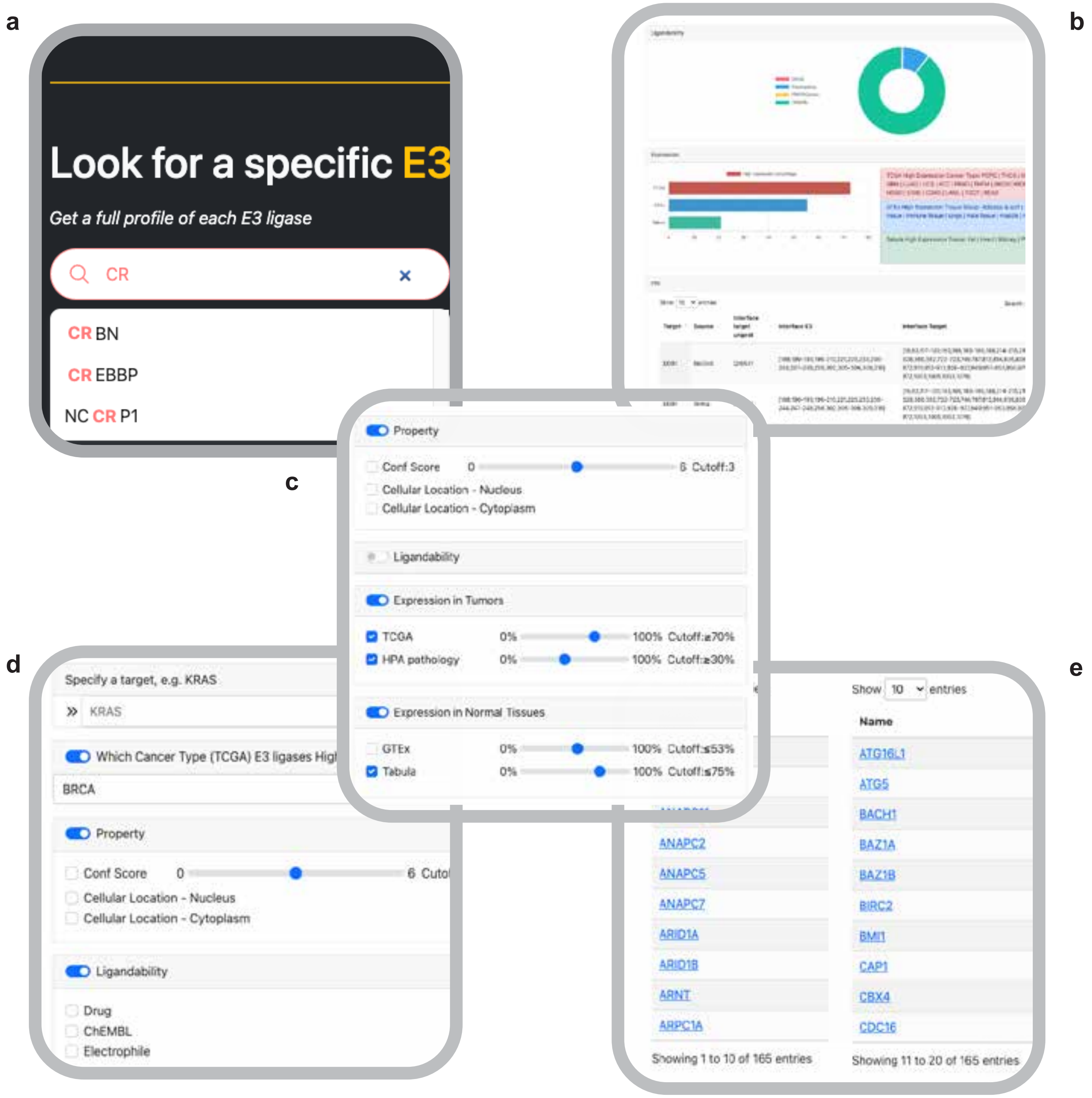

Supplementary Fig. 14 Panels of E3Atalas web portal. (a) E3 ligase profile module: directly going into a specific gene's profile. (b) comprehensive profile page of E3 ligase: showing the profile of a specific E3 ligase. (c) General E3 ligase search module: offering users the to create a set of criteria and define their own threshold. (d) Search by target module: offering users to search for E3 ligases against a specific target. (e) Search results as a table.

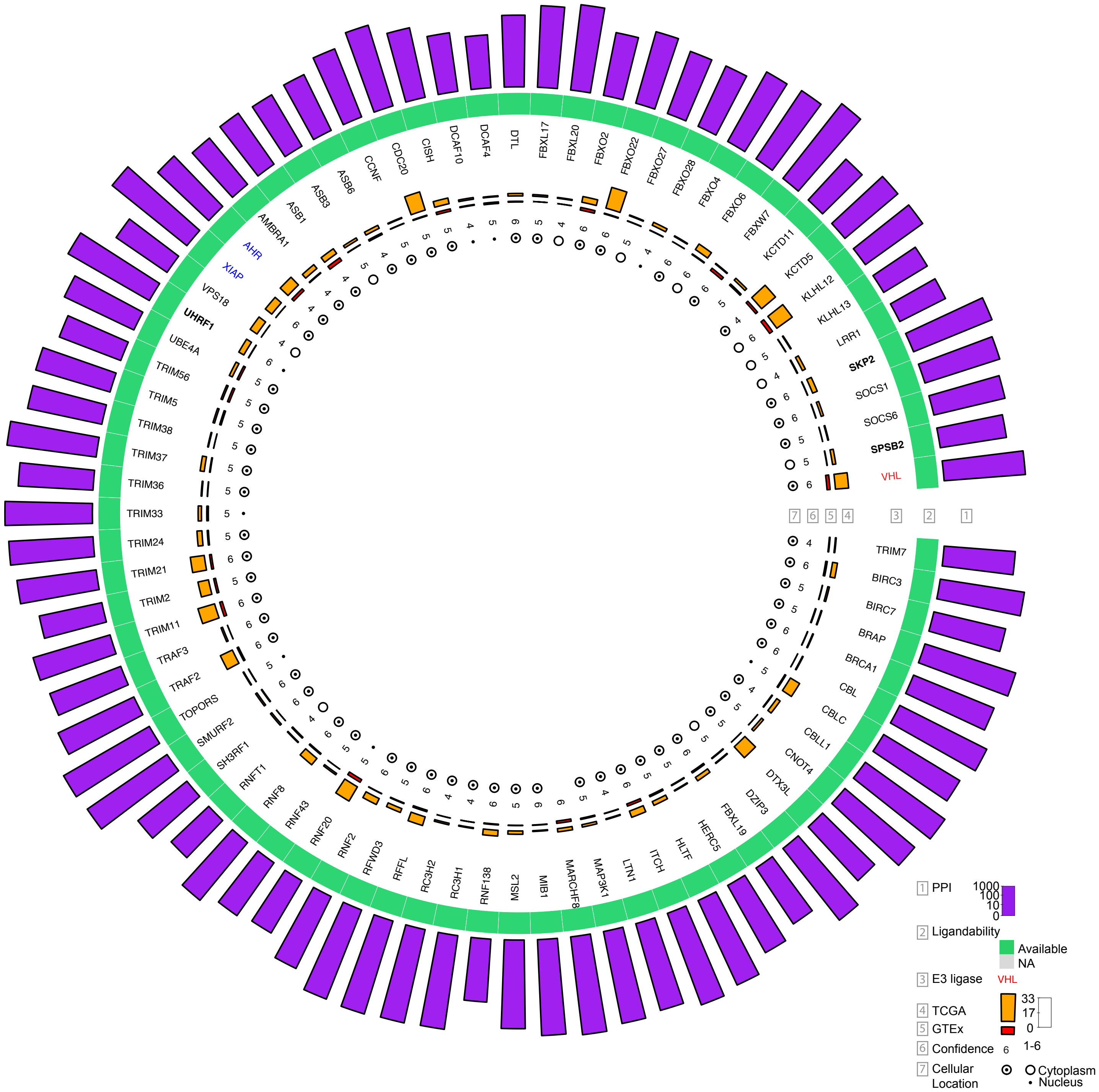

Supplementary Fig. 15 Use case: Integration of ligandability, PPI, expression pattern, confidence score, and cellular location of E3 ligases. From outer to inner ring, PPI, ligandability, E3 ligases, the number of high expression of cancer types in TCGA, the number of high expression of normal tissues in GTEx, confidence score, and cellular localization. E3 ligases in red proceeded to PROTAC clinical trials. E3 ligases in blue experimentally explored in PROTAC experiment. E3 ligases in bold were mentioned in context.

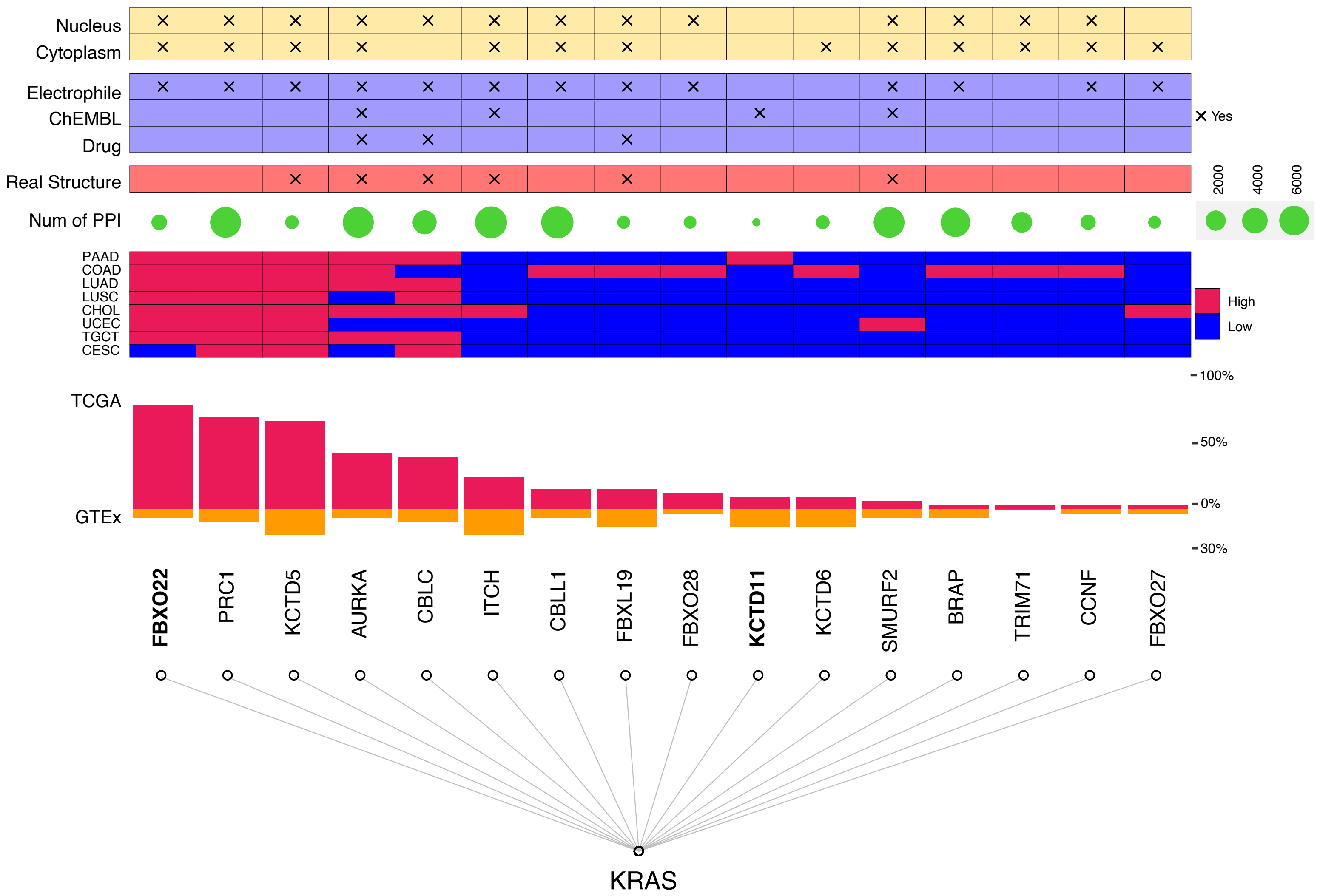

Supplementary Fig. 16 Use Case: Searching for E3 ligases against a critical oncogene, KRAS. From top to bottom: cellular location, ligandability, real structure availability, number of PPIs, expression level in PAAD, COAD, LUAD, LUSC, CHOL, UCEC, TGCT, and CESC, percentage of highly expressed cancer type in TCGA, and percentage of highly expressed normal tissues in GTEx. E3 ligases in bold were mentioned in context.

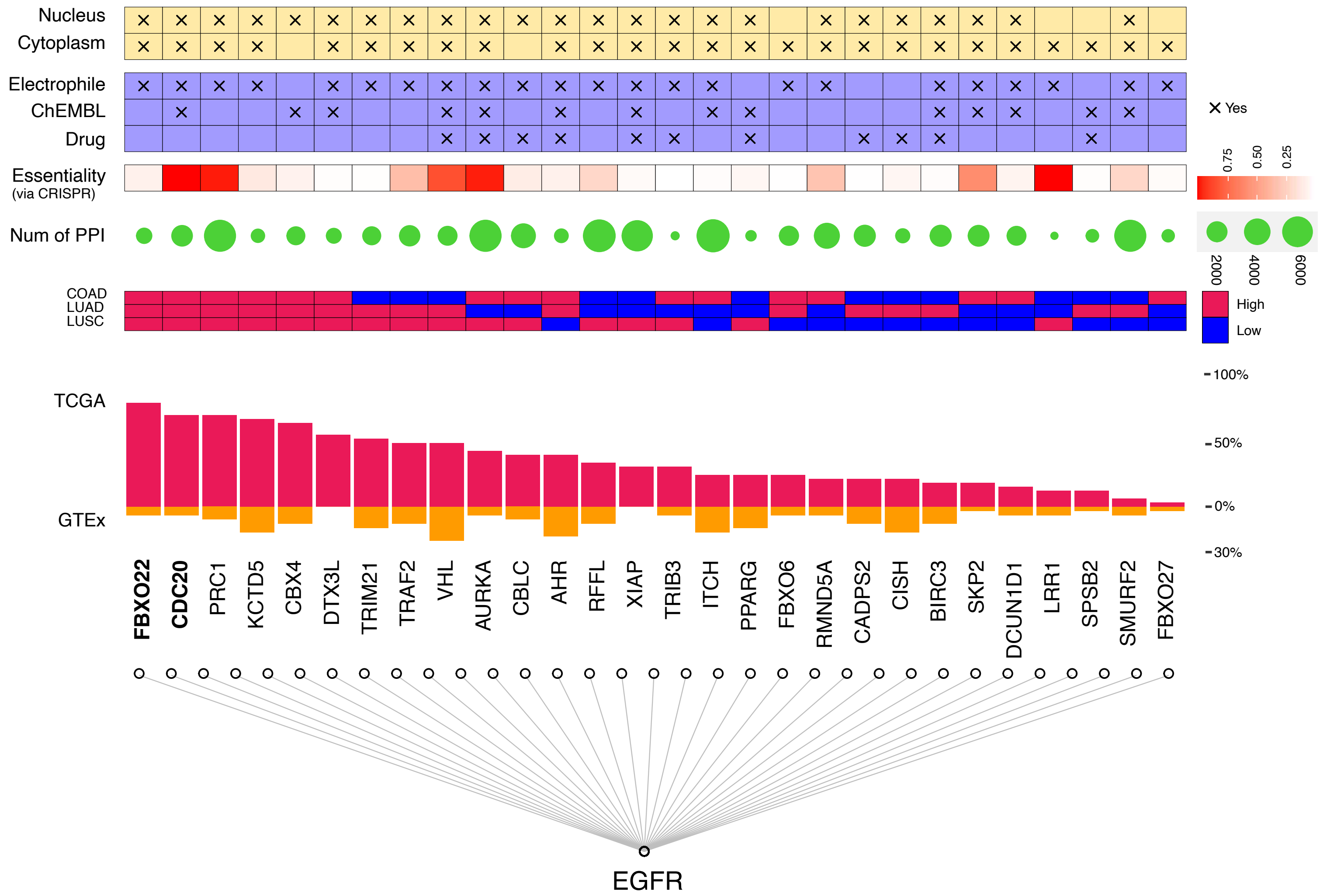

Supplementary Fig. 17 Use Case: Searching for E3 ligases against a critical oncogene, EGFR. From top to bottom: cellular location, ligandability, essentiality, number of PPIs, expression level in COAD, LUAD, and LUSC, percentage of highly expressed cancer type in TCGA, and percentage of highly expressed normal tissues in GTEx. E3 ligases in bold were mentioned in context.
