# Supplementary material for: Expanding PROTACtable genome universe of E3 ligases
Source: Nat Commun. 2023 Oct 16;14:6509. doi: 10.1038/s41467-023-42233-2 (PMC10579327; doi:10.1038/s41467-023-42233-2)
Supplement: Supplementary file 3 — Reporting Summary [file 41467_2023_42233_MOESM3_ESM.pdf]

nature portfolio

Corresponding author(s): Eytan Ruppin; Leng Han

Last updated by author(s): Sep 22, 2023

Reporting Summary

Nature Portfolio wishes to improve the reproducibility of the work that we publish. This form provides structure for consistency and transparency in reporting. For further information on Nature Portfolio policies, see our Editorial Policies and the Editorial Policy Checklist.

Statistics

For all statistical analyses, confirm that the following items are present in the figure legend, table legend, main text, or Methods section.

n/a	Confirmed	
☐	☒	The exact sample size (*n*) for each experimental group/condition, given as a discrete number and unit of measurement
☒	☐	A statement on whether measurements were taken from distinct samples or whether the same sample was measured repeatedly
☐	☒	The statistical test(s) used AND whether they are one- or two-sided<br>*Only common tests should be described solely by name; describe more complex techniques in the Methods section.*
☒	☐	A description of all covariates tested
☐	☒	A description of any assumptions or corrections, such as tests of normality and adjustment for multiple comparisons
☒	☐	A full description of the statistical parameters including central tendency (e.g. means) or other basic estimates (e.g. regression coefficient) AND variation (e.g. standard deviation) or associated estimates of uncertainty (e.g. confidence intervals)
☐	☒	For null hypothesis testing, the test statistic (e.g. *F*, *t*, *r*) with confidence intervals, effect sizes, degrees of freedom and *P* value noted<br>*Give P values as exact values whenever suitable.*
☒	☐	For Bayesian analysis, information on the choice of priors and Markov chain Monte Carlo settings
☒	☐	For hierarchical and complex designs, identification of the appropriate level for tests and full reporting of outcomes
☒	☐	Estimates of effect sizes (e.g. Cohen's *d*, Pearson's *r*), indicating how they were calculated

Our web collection on statistics for biologists contains articles on many of the points above.

Software and code

Policy information about availability of computer code

Data collection	No software was used for data collection
Data analysis	All data analysis was performed by R 4.0. Enrichment of gene ontology (GO) and Kyoto Encyclopedia of Genes and Genomes (KEGG) were tested in the clusterProfiler4.0 package. Gene symbols were mapped by human gene nomenclature (https://www.genenames.org/). Single-cell expression was processed using Seurat4.3.

For manuscripts utilizing custom algorithms or software that are central to the research but not yet described in published literature, software must be made available to editors and reviewers. We strongly encourage code deposition in a community repository (e.g. GitHub). See the Nature Portfolio guidelines for submitting code & software for further information.

Data

Policy information about availability of data

All manuscripts must include a data availability statement. This statement should provide the following information, where applicable:

- Accession codes, unique identifiers, or web links for publicly available datasets
- A description of any restrictions on data availability
- For clinical datasets or third party data, please ensure that the statement adheres to our policy

This study made use of publicly available datasets. In order to collect a comprehensive E3 ligase list, data from Ge et al., UbiHub (https://ubihub.thesgc.org/static/UbiHub.html), and UbiBrowser2.0 (http://ubibrowser.bio-it.cn) were sourced. Ligandability of E3 ligases were derived from ChEMBL (https://www.ebi.ac.uk/

chembl), DrugBank (https://go.drugbank.com), DGIdb (https://www.dgidb.org), and streamlined cysteine activity-based protein profiling (http://wren.hms.harvard.edu/cysteine_viewer). Gene expression matrices of the TCGA and GTEx were obtained in UCSC Toil Recompute Compendium (https://xenabrowser.net). Protein expression levels in tumors were downloaded from HPA pathology atlas (https://www.proteinatlas.org/humanproteome/pathology). Single-cell transcriptomics data in normal samples were fetched from the Tabula Sapiens portal (https://tabula-sapiens-portal.ds.czbiohub.org), and data in tumors were acquired in the TISCH database (http://tisch1.comp-genomics.org). Matched public single-cell expression of tumor samples were originally from GSE143423(BRCA, https://www.ncbi.nlm.nih.gov/geo/query/acc.cgi?acc=GSE143423), EMTAB6149(NSCLC, https://www.ebi.ac.uk/biostudies/arrayexpress/studies/E-MTAB-6149), GSE139829 (UVM, https://www.ncbi.nlm.nih.gov/geo/query/acc.cgi?acc=GSE139829), CRA001160 (PAAD, https://ngdc.cncb.ac.cn/gsa/browse/CRA001160), GSE138794 (Glioma, https://www.ncbi.nlm.nih.gov/geo/query/acc.cgi?acc=GSE138794). PPIs and ESIs were collected from BioGrid (https://thebiogrid.org), IntAct (https://www.ebi.ac.uk/intact), Reactome (https://reactome.org), HuRI (http://www.interactome-atlas.org), STRING (https://string-db.org), and UbiBrowser2.0 (http://ubibrowser.bio-it.cn). The role of target gene in cancer was annotated with NCG (http://ncg.kcl.ac.uk/). Protein-protein interaction interfaces was downloaded from InteractomeInsider (http://interactomeinsider.yulab.org/). Structures of E3 ligases were queried and summarized from the PDB (https://www.rcsb.org/) and AlphaFoldDB (https://alphafold.ebi.ac.uk/). Essentiality of genes in tumors were obtained from the DepMap via CRISPR method (https://depmap.org/portal) and an RNAi-based study. Cellular locations were determined using the Gene Ontology database (http://geneontology.org), COMPARTMENTS (https://compartments.jensenlab.org), and UniProt (https://www.uniprot.org). Processed data was deposited into our web portal (https://hanlaboratory.com/E3Atlas).

Research involving human participants, their data, or biological material

Policy information about studies with human participants or human data. See also policy information about sex, gender (identity/presentation), and sexual orientation and race, ethnicity and racism.

Reporting on sex and gender	N/A
Reporting on race, ethnicity, or other socially relevant groupings	N/A
Population characteristics	N/A
Recruitment	N/A
Ethics oversight	N/A

Note that full information on the approval of the study protocol must also be provided in the manuscript.

Field-specific reporting

Please select the one below that is the best fit for your research. If you are not sure, read the appropriate sections before making your selection.

☒ Life sciences ☐ Behavioural & social sciences ☐ Ecological, evolutionary & environmental sciences

For a reference copy of the document with all sections, see nature.com/documents/nr-reporting-summary-flat.pdf

Life sciences study design

All studies must disclose on these points even when the disclosure is negative.

Sample size	In this study, we downloaded all tumor samples of The Cancer Genome Atlas (TCGA) (N = 9785) and all normal samples of Genotype-Tissue Expression (GTex) (N = 7862) from UCSC Toil Recompute Compendium (https://xenabrowser.net). Protein expression patterns in cancer tissues (216 tumors) were obtained from from HPA pathology atlas (https://www.proteinatlas.org/humanproteome/pathology). Single-cell transcriptomics data in normal samples from 15 patients were fetched from the Tabula Sapiens portal (https://tabula-sapiens-portal.ds.czbiohub.org). Matched public single-cell expression of tumor samples were originally from GSE143423(BRCA, N=1, https://www.ncbi.nlm.nih.gov/geo/query/acc.cgi?acc=GSE143423), EMTAB6149(NSCLC, N=5, https://www.ebi.ac.uk/biostudies/arrayexpress/studies/E-MTAB-6149), GSE139829 (UVM, N=11, https://www.ncbi.nlm.nih.gov/geo/query/acc.cgi?acc=GSE139829), CRA001160 (PAAD, N=35, https://ngdc.cncb.ac.cn/gsa/browse/CRA001160), GSE138794 (Glioma, N=9, https://www.ncbi.nlm.nih.gov/geo/query/acc.cgi?acc=GSE138794).
Data exclusions	When selecting single-cell transcriptomics of tumor samples in TISCH, studies were excluded if it does not meet the following criteria: (1) sampling from humans; (2) treatment naïve; (3) assayed via 10X Genomics; (4) malignant cells annotated. When multiple studies shared with the same cancer type, the study with the largest number of samples was retained for the subsequent analysis.
Replication	All data analyses were reproducible.
Randomization	Samples were analyzed by cancer type or tissue type, and no randomization were performed.
Blinding	All data was obtained from public data resources, so blinding was not relevant.

Reporting for specific materials, systems and methods

We require information from authors about some types of materials, experimental systems and methods used in many studies. Here, indicate whether each material, system or method listed is relevant to your study. If you are not sure if a list item applies to your research, read the appropriate section before selecting a response.

Materials & experimental systems

n/a	Involved in the study
☒	☐ Antibodies
☒	☐ Eukaryotic cell lines
☒	☐ Palaeontology and archaeology
☒	☐ Animals and other organisms
☒	☐ Clinical data
☒	☐ Dual use research of concern
☒	☐ Plants

Methods

n/a	Involved in the study
☒	☐ ChIP-seq
☒	☐ Flow cytometry
☒	☐ MRI-based neuroimaging
