# Supplementary material for: Expanding PROTACtable genome universe of E3 ligases
Source: Nat Commun. 2023 Oct 16;14:6509. doi: 10.1038/s41467-023-42233-2 (PMC10579327; doi:10.1038/s41467-023-42233-2)
Supplement: Supplementary file 4 — Description of Additional Supplementary Files [file 41467_2023_42233_MOESM4_ESM.pdf]

Description of Additional Supplementary Files

File Name: Supplementary Data 1.xlsx
Description: Collected E3 ligases from three distinct sources
